# A mycobacterial DivIVA domain-containing protein involved in cell length and septation

**DOI:** 10.1099/mic.0.000952

**Published:** 2020-07-17

**Authors:** Hayleah Pickford, Emily Alcock, Albel Singh, Gabriella Kelemen, Apoorva Bhatt

**Affiliations:** ^1^​ School of Biosciences and Institute of Microbiology and Infection, University of Birmingham, UK; ^2^​ School of Biological Sciences, University of East Anglia, Norwich, UK

**Keywords:** *Mycobacterium*, septation, division, cytoskeleton, polar growth, tuberculosis

## Abstract

Mycobacterial cells elongate via polar deposition of cell wall material, similar to the filamentous *Streptomyces* species, which contain a tip-organizing centre. Coiled-coiled proteins such as DivIVA play an important role in this process. The genome of *Mycobacterium tuberculosis *, the causative agent of tuberculosis, encodes many coiled-coil proteins that are homologous to DivIVA with a potential role in mycobacterial cell elongation. Here we describe studies on *Mycobacterium smegmatis MSMEG_2416*, a homologue of *M. tuberculosis Rv2927c*. Two previous independent studies showed that *MSMEG_2416* was involved in septation (subsequently referred to as *sepIVA*). Contrary to these previous reports, we found *sepIVA* to be dispensable for growth in laboratory media by generating a viable null mutant. The mutant strain did, however, show a number of differences, including a change in colony morphology and biofilm formation that could be reversed on complementation with *sepIVA* as well as *Rv2927c*, the *sepIVA* homologue from *M. tuberculosis *. However, analysis of cell wall lipids did not reveal any alterations in lipid profiles of the mutant strain. Microscopic examination of the mutant revealed longer cells with more septa, which occurred at irregular intervals, often generating mini-compartments, a profile similar to that observed in the previous studies following conditional depletion, highlighting a role for *sepIVA* in mycobacterial growth.

## Introduction

A majority of widely studied rod-shaped bacteria such as *Escherichia coli* and *Bacillus subtilis* elongate by the lateral deposition of new cell wall material along the whole length of the bacterium. It is the highly regulated septal positioning and formation that results in the formation of morphologically identical daughter cells. The essential bacterial tubulin homologue FtsZ [1] is crucial in the initiation of a contractile protofilament ring, the Z ring, at the site of septum formation. The Min system, active and highly studied in both *E. coli* and *B. subtilis *, is composed of a number of interacting proteins that inhibit Z ring formation at sites distant from the mid cell, resulting in septum formation at a defined range at the centre of the cell [2, 3]. *B. subtilis* possesses a septum-determining protein termed DivIVA [4], which recognizes membranes of negative curvature [5], hence its localization at the cell poles, and the septum during the initiation of septation [4]. DivIVA is responsible for the maintenance of a high concentration of the FtsZ inhibitor, MinC, at the cell poles, ensuring correct positioning of septum formation at the mid cell [3].

Members of the genus *Mycobacterium *, including the tuberculosis-causing *Mycobacterium tuberculosis *, exhibit polar growth with newly synthesized cell wall deposited at the poles of the rod-shaped bacterium in contrast to the lateral deposition seen in *Bacillus* and other bacteria. Mycobacteria do not possess *min* homologues, yet they do possess a *divIVA* homologue, termed *wag31*. Depletion of Wag31 in the fast-growing saprophyte *Mycobacterium smegmatis* produced cells that were rounded at one pole, progressively becoming more coccoid in shape, and eventually, lysing [6]. Depletion of Wag31 resulted in reduced Wag31 localization at the cell poles, with the subsequent inability to direct nascent peptidoglycan synthesis at the poles [6]. The outer section of the mycobacterial cell wall consists of a lipid-rich layer that contain unique lipids, including mycolic acids [7], and Wag31 is also known to recruit enzymes involved in the biosynthesis of these lipidic components to poles of growing cells [8–11].

The filamentous actinomycete, *Streptomyces coelicolor *, also exhibits polar growth; the DivIVA homologue in *S. coelicolor* interacts with a number of coiled-coil proteins, including the filament-forming protein FilP [12], and a novel protein termed Scy [13]. Scy controls the number and location of tips formed through the sequestering of cell wall synthesis, via its interaction with DivIVA [13]. These three interacting proteins are constituents of the *Streptomyces* tip-organizing centre (TIPOC), a multi-protein complex essential for polarized growth in this genus.

It is likely that mycobacteria also encode components of a TIPOC that drives polar growth, similar to that in *Streptomyces *. The *M. tuberculosis* genome contains a number of genes that potentially encode coiled-coil proteins similar to FilP and Scy. This study focuses on a putative coiled-coil protein encoded by *M. smegmatis *, *MSMEG_2416,* a homologue of the *M. tuberculosis* gene *Rv2927c*.

Two previous independent studies by Wu *et al*. [14] and Jain *et al*. [15] showed that *MSMEG_2416* encodes a coil-coiled protein containing a DivIVA-like domain that associated with the septum in the later stages of cell division, localizing to the division site [14]. The gene was termed *sepIVA* and was first identified as a gene encoding a protein that interacts with another mycobacterial septal factor, FtsQ [14, 15]. Neither study was able to generate a viable *M. smegmatis sepIVA* null mutant, suggesting gene essentiality, and instead used recombinant strains that allowed conditional depletion of SepIVA to study function. Depletion of the septal factor led to elongated cells that failed to divide and formed branched filaments [14]. SepIVA also appeared to migrate from the septum to the intracellular membrane domain (IMD), as sub-polar site thought to be a focal point for enzymes involved in the biosynthesis of cell components [16], suggesting that IMD was a site for ‘reserve’ SepIVA, or that the protein had additional, yet unknown, functions [14].

Contrary to these two reports of the essentiality of *sepIVA*, we were able to generate a null mutant of *sepIVA* on laboratory media. Here we describe the functional characterization of the *M. smegmatis sepIVA* null mutant strain and discuss potential reasons for our ability to generate a null mutant despite earlier reports of essentiality [17].

## Results

### Homologues of *sepIVA* in other bacteria

**Fig. 1.**
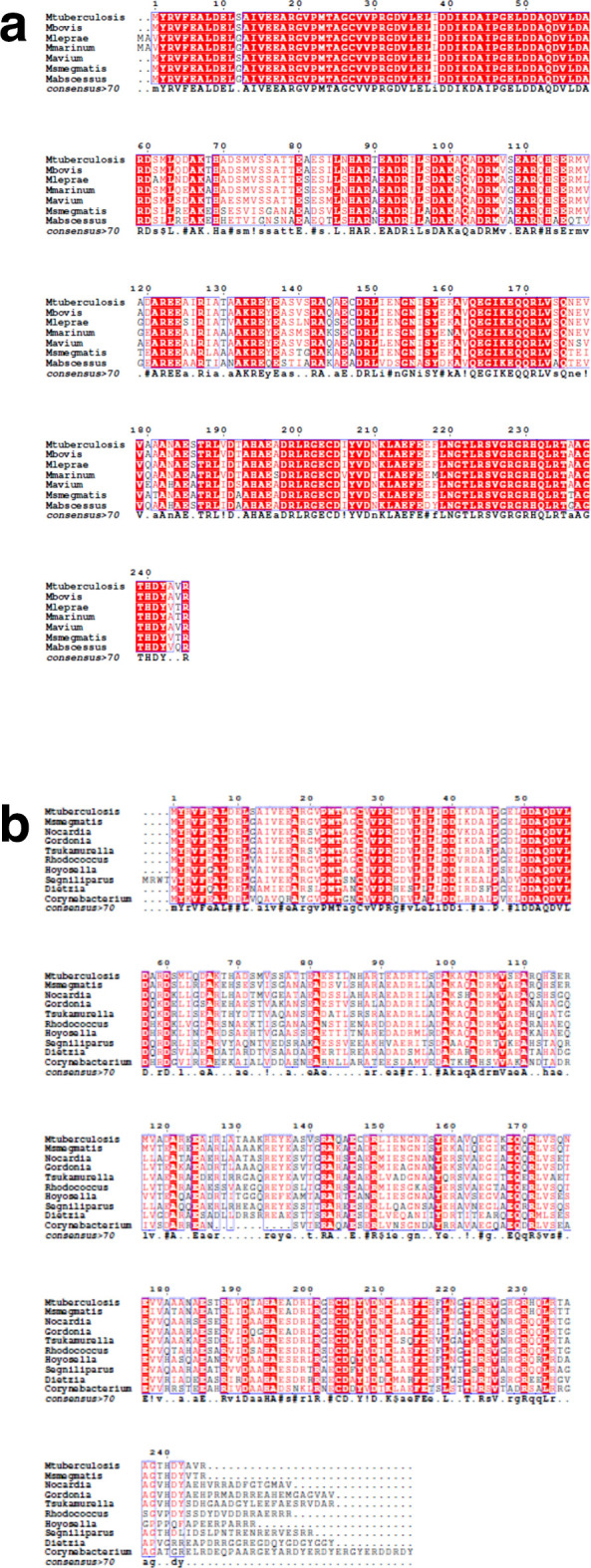
Alignment of the *M. smegmatis* SepIVA amino acid sequence with homologues in other mycobacterial species (a) and with genera from the suborder *Corynebacterianea* (b).

To determine if *sepIVA* was a core mycobacterial gene, we first searched for homologues in other mycobacterial genomes. Homologues of *sepIVA* were found in the decayed genome of *Mycobacterium leprae *, in environmental mycobacteria and in other members of the *Mycobacterium tuberculosis* complex, indicating the presence of this *divIVA*-like gene across mycobacterial species (Fig. 1a). However, surprisingly, we also found homologues of *sepIVA* across members of *Corynebacterianea*, a suborder of the *Actinomycetales *, consisting of various mycolate-producing genera (Fig. 1b). Homologues, albeit with lower similarity/identity scores, were also found in other suborders of the *Actimomycetales*, including the filamentous *Streptomyces* sp., which also exhibit polar growth (data not shown). No significant matches were revealed in blastp searches of genomes of bacterial genera outside of this group, including *Bacillus subtilis* and *Escherichia coli *. These findings suggested that *sepIVA* represented conserved coiled-coil proteins that were present in ancestral polar growth progenitors of the *Actinomycetales *.

### Essentiality of *sepIVA* in *M. smegmatis*

To probe the role of *sepIVA* in mycobacterial growth, we aimed to generate *M. smegmatis* cells that were depleted of *sepIVA* function. We commenced this study prior to the publication of the two aforementioned studies on *M. smegmatis sepIVA*, and at the time were guided solely by the predicted essentiality of the *M. tuberculosis sepIVA* gene (*Rv2927c*) [17], anticipating *sepIVA* to be an essential gene in *M. smegmatis *. However, prior to using a gene essentiality testing tool [18] to study cells conditionally depleted of *sepIVA*, we first attempted to generate a knockout of *sepIVA* in *M. smegmatis* to validate a potential inability to delete *sepIVA* in a wild-type (WT) strain. We transduced *M. smegmatis* mc^2^155 with phΔ*MSMEG2416*, a recombinant phage designed to replace *sepIVA* with a hygromycin resistance cassette (*hyg*) by specialized transduction [19]. Surprisingly, we were able to generate hygromycin-resistant transductants, indicating a replacement of *sepIVA* with *hyg* in the WT strain. One such transductant was analysed by whole-genome sequencing, confirming the replacement of *sepIVA* with *hyg* (Fig. S1, available in the online version of this article). The transductant was selected for further analysis and is referred to as Δ*sepIVA*. The ability to generate a viable *sepIVA* mutant in WT *M. smegmatis* demonstrated that the gene was not essential for the viability and growth of *M. smegmatis* in laboratory media, which in this case was tryptic soy broth (TSB) agar. We further investigated possible factors that may have affected our ability to obtain a viable *sepIVA* mutant, contrary to the earlier reports of essentiality. The two previous studies used 7H9 and 7H10 for growth [14, 15], while we used TSB agar to select for transductants. To probe potential effects of media, we tested the ability of the Δ*sepIVA* strain to grow on plates of three Middlebrook media, 7H9+agar, 7H10 and 7H11, compared to TSB agar. While the mutant strain formed smaller colonies on the Middlebrook media plates, we did not observe any significant changes in the efficiency of obtaining colony-forming units (c.f.u.) between the different agar media, suggesting that the media used for the generation of recombinant strains did not play a role in the differing outcome of our study (Fig. S2). Next, we explored the possibility that a second, pre-existing mutation in a fraction of the transduced *M. smegmatis* cells may have enabled us to isolate a viable *sepIVA* mutant. Synthetic lethality can lead to an inability to generate knockouts of otherwise non-essential genes unless the lethality is abrogated by the concurrent loss of a second gene function. This has been observed in genes involved in the α-glucan pathway of *M. tuberculosis* [20]. Using the whole-genome sequence, we performed a variant call analysis to compare single-nucleotide polymorphisms (SNPs) of the parental WT *M. smegmatis* strain and the Δ*sepIVA* mutant strain, comparing both to the reference genome sequence of *M. smegmatis* mc^2^155 (NC_008596.1). One particular SNP, in a gene required for septation, stood out from the list of SNPs found only in the Δ*sepIVA* strain: a single nucleotide change in the gene *ftsW* that resulted in the change of an aspartate residue at position 91 to an asparagine. FtsW is required for septation and for the mid-cell positioning of penicillin-binding protein 3 (PBP3) [21]. In an interaction unique to mycobacteria, FtsW also forms a ternary complex that includes FtsZ and PBP3 with a potential role in septal peptidoglycan synthesis [21]. Given its association with septation, we reckoned that if a pre-existing mutated *ftsW* gene rendered the resident cell amenable to the generation of a viable *sepIVA* mutant, expression of a WT copy of *ftsW* in the mutant strain would be lethal. However, we were able to obtain viable transformants when an integrating plasmid carrying *M. smegmatis ftsW* with its native promoter was electroporated into the Δ*sepIVA* strain, suggesting that the mutation in *ftsW* was unlikely to have influenced our ability to generate a viable *sepIVA* knockout in *M. smegmatis* (one such transformant is shown in Fig. S3). Our recombinant phage was designed to replace residues 466–602 of the 738 bp *sepIVA* gene with the *hyg-sacB* cassette from the allelic exchange vector, thus retaining a substantial section of the 5′ end of *sepIVA* open reading frame after allelic exchange that resulted in the knockout strain. It is possible that if there was a readthrough into the replacement cassette after allelic exchange, a shorter peptide containing the first 155 amino acids of the N-terminus of SepIVA (246 aa long) would be produced, and subsequently aided the formation of a viable mutant strain. Using web-based translation tools [22] we were able to test the sequences obtained from the allelic exchange vector and predicted a 174 aa long peptide retaining 155 aa from the N-terminus of SepIVA to be formed. Due to a lack of antibodies against SepIVA, we do not have evidence that this peptide is produced in our Δ*sepIVA* strain, but the phenotypes displayed by our mutant strain (described below) suggest that while this putative shorter SepIVA may have enabled us to generate a viable knockout strain, it did not retain full functionality.

### Deletion of *sepIVA* alters colony morphology and biofilm formation

**Fig. 2.**
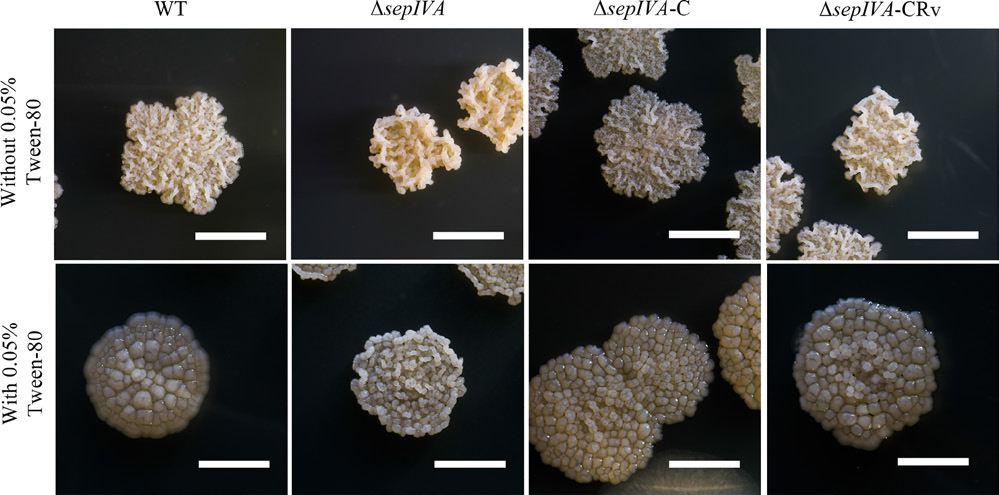
Colonies of *M. smegmatis* WT, Δ*sepIVA*, Δ*sepIVA-*C and Δ*sepIVA*-CRv strains grown on TSB agar. Scale bar, 5 mm.

**Fig. 3.**
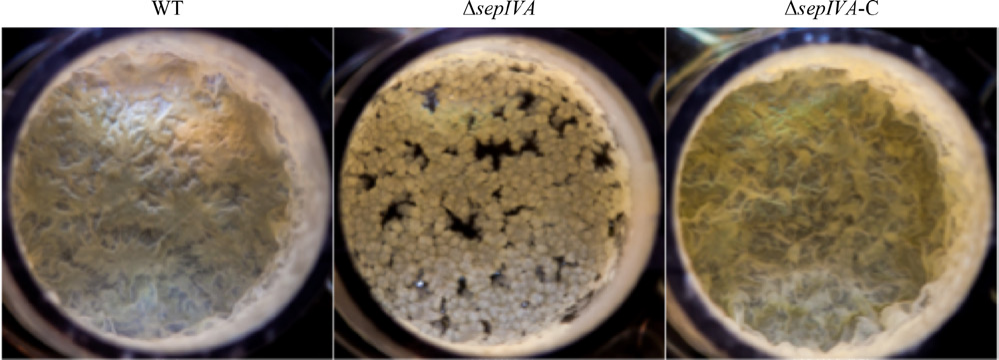
Pellicles of *M. smegmatis* wild-type (WT), Δ*sepIVA* and Δ*sepIVA*-C strains cultured in 24 well plates containing Sauton’s media (each well has a diameter of 15 mm). The pictures represent one of two repeats.

**Fig. 4.**
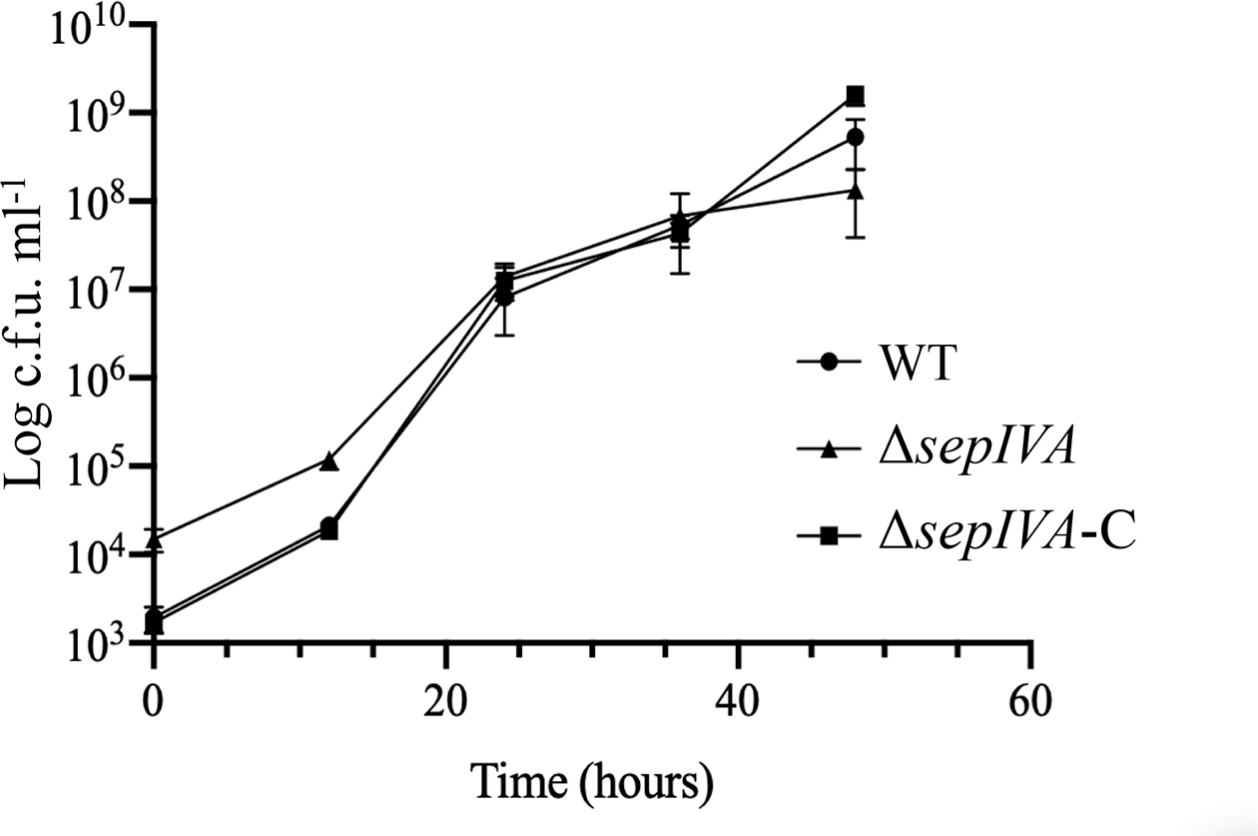
Growth curve of *M. smegmatis* WT, *ΔsepIVA* and Δ*sepIVA*-C strains in TSB supplemented with 0.05 % Tween-80, and grown at 37 °C with agitation.

While we were able to generate a null mutant of *sepIVA* in *M. smegmatis *, there was a striking difference in the appearance of colonies of Δ*sepIVA* as compared to the parental (wild-typeWT) strain, *M. smegmatis* mc^2^155 (Fig. 2). Colonies of the Δ*sepIVA* strain appeared ‘drier’ and more convoluted compared to the WT strain, with the phenotype more apparent when cultured on tryptic soy agar supplemented with 0.05 % Tween-80 (Fig. 2). Introduction of an integrating plasmid-borne copy of *sepIVA* with its native promoter into Δ*sepIVA* restored colony morphology to that of the WT strain, indicating that the observed changes were solely due to the loss of *sepIVA*. Colony morphology could also be restored in Δ*sepIVA* transformed with a plasmid-borne copy of the *M. tuberculosis sepIVA* (*Rv2927c*), indicating that the *M. tuberculosis* homologue could functionally complement the Δ*sepIVA* mutant. The mutant also demonstrated an impaired ability to form air–water interface biofilms (pellicles) (Fig. 3). In spite of these differences of growth morphologies, we surprisingly observed no changes in the growth rates of the Δ*sepIVA* strain when compared to the WT and complemented strains (Fig. 4).

### Cell wall lipid profiles of the Δ*sepIVA* mutant

**Fig. 5.**
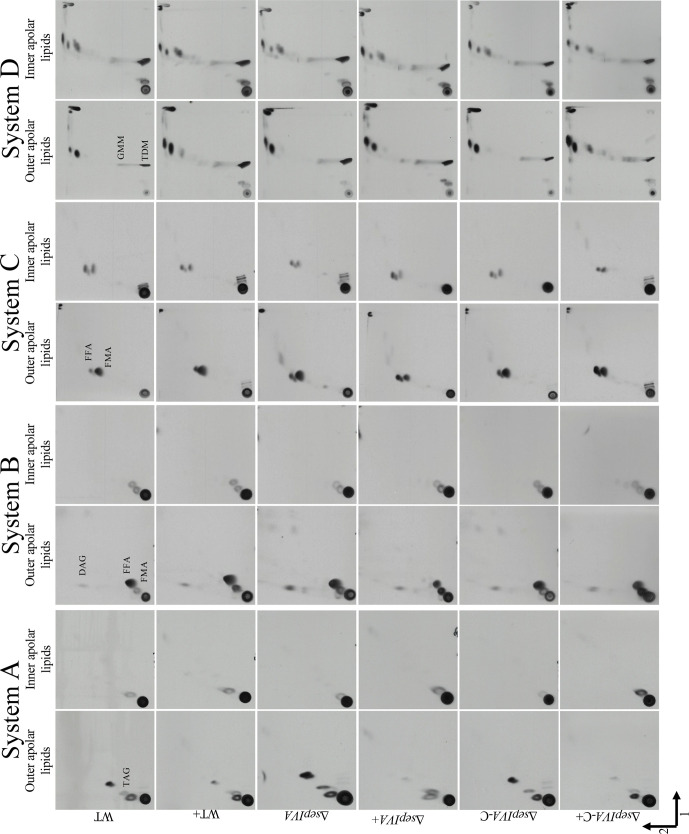
2D TLC of apolar lipids extracted from *M. smegmatis* WT, Δ*sepIVA* and Δ*sepIVA*-C strains grown on TSB agar containing [^14^C]-acetic acid at 37 °C for 7 days. The presence of Tween-80 on the plates is indicated by a ‘+’. Solvent systems A–D are as described [28]. TAG, triacylated glycerol; DAG, diacylated glycerol; TDM, trehalose monomycolate; GMM, glucose monomycolate; FFA, free fatty acids, FMA, free mycolic acids.

**Fig. 6.**
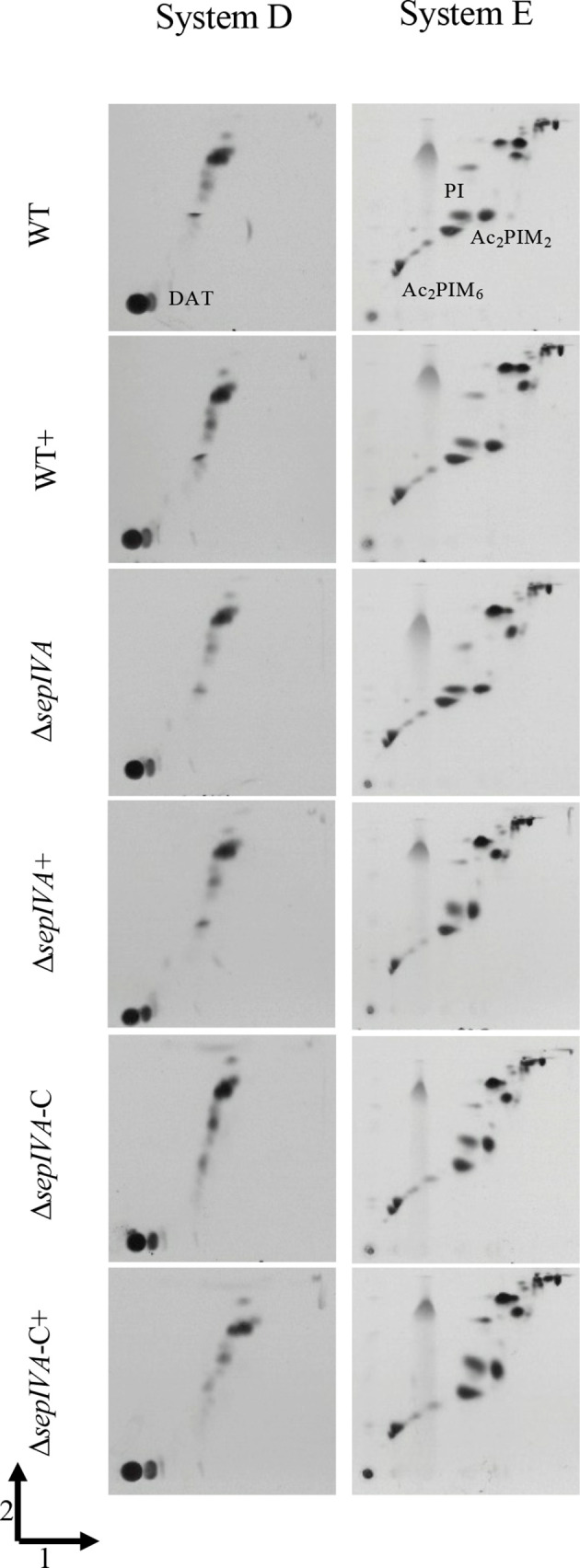
2D TLC of polar lipids extracted from *M. smegmatis* WT, Δ*sepIVA* and Δ*sepIVA*-C strains grown on TSB agar containing [^14^C]-acetic acid at 37 °C for 7 days. The presence of Tween-80 on the plates is indicated by a ‘+’. Solvent systems D and E are as described [28]. DAT, diacylated trehalose; PI, Ac_2_PIM_2_ and AC_2_PIM_6_ indicate phospholipids.

Alterations in the outer cell wall lipids in mycobacteria are often responsible for changing colony morphology [23, 24]. Also, cell wall lipid biosynthesis enzymes are known to interact with the cytoskeletal machinery to co-ordinate cell wall biogenesis with polar growth [6, 25]. To probe changes in cell wall lipids in the mutants, we grew colonies of the WT, mutant and complemented strains on agar plates with [^14^C]-acetic acid to label cell wall lipids. Apolar and polar fractions of the lipids were extracted from scraped colonies and analysed by two-dimensional thin-layer chromatography (2D TLC) (Figs 5 and 6). Surprisingly, no alterations in lipid profiles were seen in the mutant strain, indicating that the altered colony morphology was not caused by a change in cell wall lipid composition.

### Loss of *sepIVA* affects average cell length

**Fig. 7.**
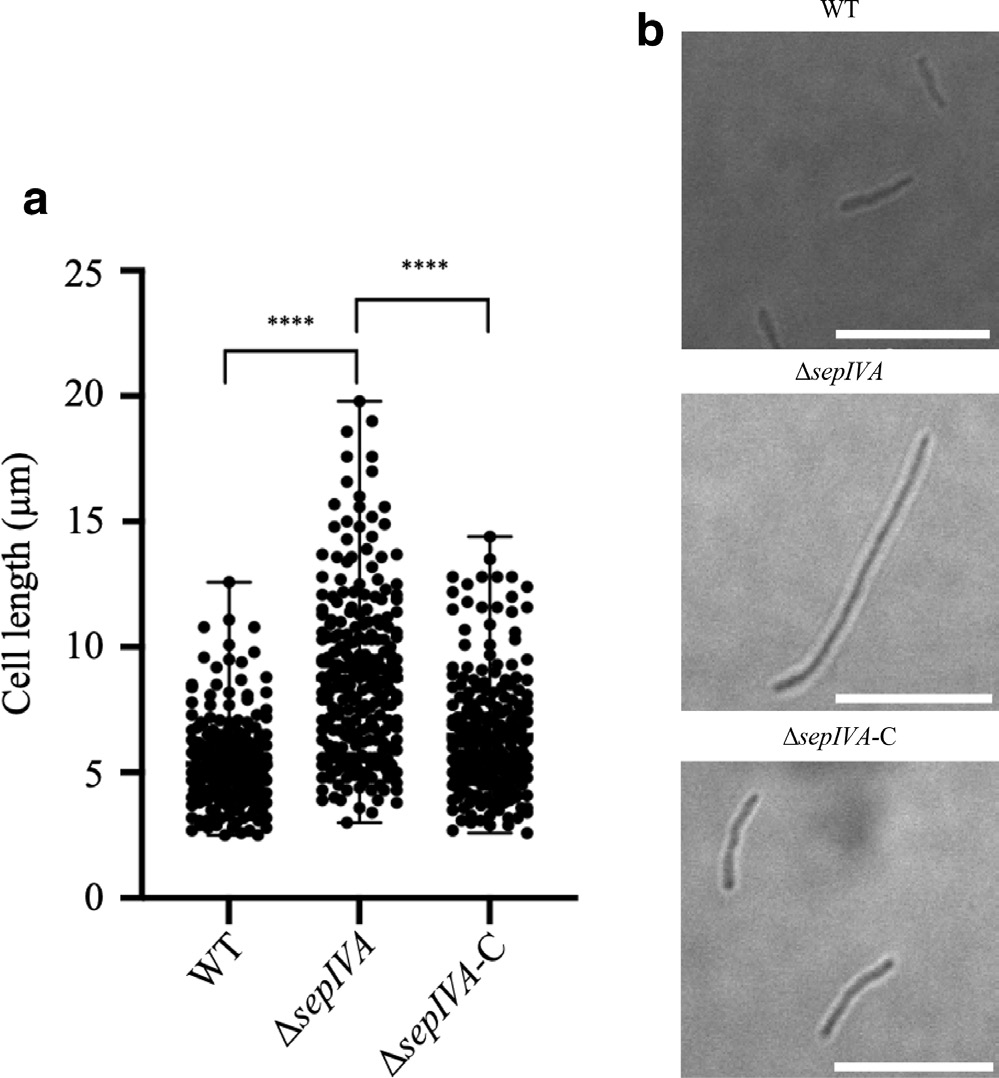
Microscopy and cell length analysis of WT *M. smegmatis,* Δ*sepIVA* and Δ*sepIVA*-C. Strains were cultured in TSB supplemented with 0.05 % Tween-80, and visualized at 100× magnification. (a) A random sample of 250 cells from mid-exponential phase cultures (two biological repeats) were measured to determine average cell lengths. The deletion of *sepIVA* resulted in the production of significantly longer cells (b), when compared to WT *M. smegmatis* and Δ*sepIVA*-C. ****, *P*<0.0001. Scale bar, 10 µM.

To further probe the effects of loss of *sepIVA*, we observed mid-log phase cultures of the mutant strain using light microscopy, comparing them to those of the WT strain. Cells of the Δ*sepIVA* strain were significantly longer (*P*<0.05), when compared to the WT and complemented strains (Fig. 7). The average length of cells of the mutant strain was 8.8 µm compared to 5.4 µm for the WT strain and 6.5 µm for the complemented strain, suggesting that loss of *sepIVA* affected average cell length, possibly due to defects in division.

### Loss of *sepIVA* leads to altered septation patterns

**Fig. 8.**
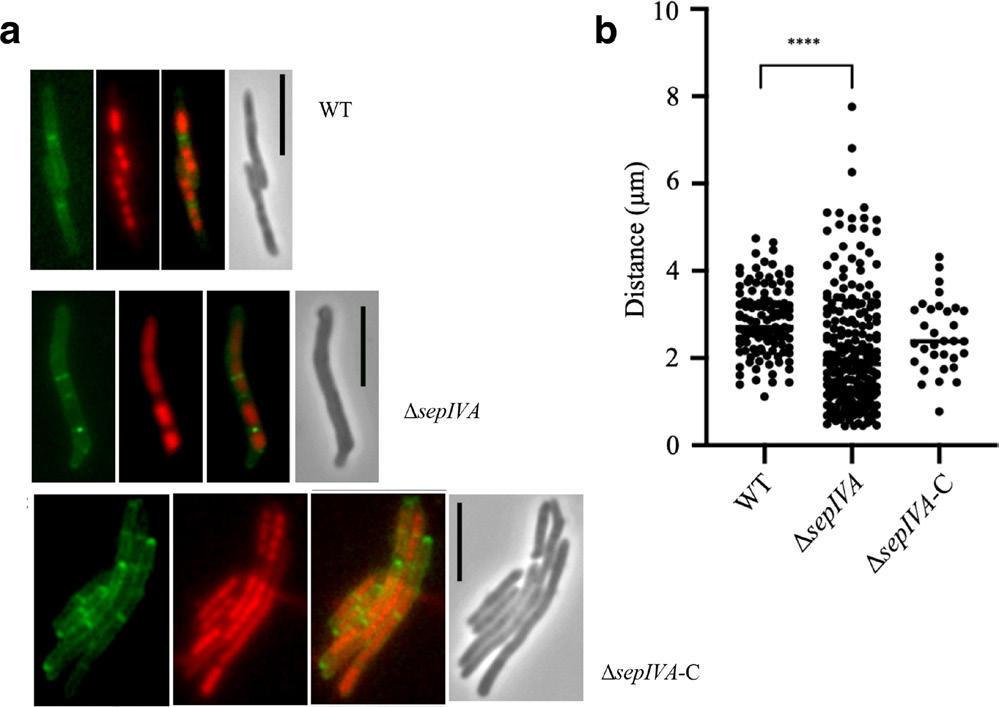
Fluorescence microscopy and analysis of septation in WT *M. smegmatis,* Δ*sepIVA* and Δ*sepIVA*-C. Strains were cultured to mid-exponential phase in TSB supplemented with 0.05 % Tween-80, and the cell membrane and chromosomal matter was stained using fluorescent vancomycin (green) and propidium iodide (red), respectively, and one representative image is shown for each strain (a). The distance between septa of a population of cells was recorded and analysed statistically (b). A total of 116, 232 and 32 septal distance measurements (septum to septum, or septum to cell end) were made for WT, Δ*sepIVA* and Δ*sepIVA*-C, respectively (two biological replicates). *SepIVA* deletion resulted in aberrant septation, with the distance between septa to septa or cell end being statistically shorter than for WT *M. smegmatis *. ****, *P*<0.0001.

To further query the long cell phenotype we observed in the Δ*sepIVA* strain, we investigated the formation of septa in the mutant strain using fluorescent vancomycin staining. The mutant strain showed an irregular, more frequent septation pattern when compared to the WT strain; the longer cells of Δ*sepIVA* had more cross walls and often generated mini-compartments (Fig. 8). Septation phenotypes were restored to WT patterns in the complemented strain. These findings showed that loss of *sepIVA* dysregulated patterns of septum formation in *M. smegmatis *.

## Discussion

Mycobacteria require both common and bespoke cytoskeletal proteins that drive the distinct polar growth pattern seen in this genus and other related bacterial genera, such as *Streptomyces *. At the start of the study described here we were interested in probing the function of *MSMEG_2416* (termed *sepIVA*, and a homologue of *M. tuberculosis Rv2927c*), a gene encoding a coiled-coiled protein that shares a domain with the septum forming protein DivIVA. We were able to generate a viable null mutant of *M. smegmatis sepIVA* to help us address the role of this gene in mycobacterial growth. During the course of this study, two independent reports on mycobacterial septal factors were published [14, 15], outlining a role for *sepIVA* in septum formation in mycobacterial cells. Contrary to our studies, both studies reported *sepIVA* as an essential gene in *M. smegmatis *. We thus continued our studies and characterized the *M. smegmatis sepIVA* mutant we had generated. Deletion of *sepIVA* also seemed to produce longer cells and affected the culture characteristics of the strain, including the formation of altered colony morphology. It also affected septation patterns in the mutant strain, which produced irregular septation.

Similar to our approach, Jain *et al*. used specialized transduction for allelic exchange of the native copy of *sepIVA*, but using a FLAS-tagged, Tet-regulated second copy of *sepIVA* in a merodiploid strain [15]. Wu *et al*., on the other hand, were unable to generate a conditional mutant using an approach that involved generating a merodiploid with *sepIVA* under the control of a non-native promoter. Instead they engineered a strain containing a DAS-tag at the C-terminus of SepIVA. The DAS-tag made SepIVA amenable to conditional depletion by controlled degradation, which led to the formation of filamentous cells [14]. It is unlikely that downstream effects on *MSMEG_2417* might have led to a different outcome of essentiality in these two studies, as *MSMEG_2417* is not essential in *M. smegmatis* [26]. Moreover, the conditional mutants described in these studies showed similar phenotypes to WT *M. smegmatis* under conditions that allowed for expression of SepIVA function [14, 15]. *Rv2927c* is predicted to be essential in *M. tuberculosis *, but *M. smegmatis* can tolerate the loss of genes known to be essential in *M. tuberculosis *. Moreover, a study by Dragset *et al*. [26] identified an *in vitro* gene essentiality set for *M. smegmatis* that indicated that *sepIVA* is a non-essential gene.

We queried various reasons likely to explain the contrast between our report of non-essentiality of *sepIVA* and those of *sepIVA* essentiality in the two previous studies [14, 15]. We ruled out the role of growth media, as the mutant generated in this study was able to grow on the media used in the previous reports. We did identify a SNP in another cell division-associated gene, *ftsW*, and queried its role in the viability of our Δ*sepIVA* mutant. We postulated that if the WT copy of *ftsW* was dominant, its introduction into the Δ*sepIVA* strain would cause lethality. However, the transformants obtained after electroporation of Δ*sepIVA* were viable after plating on selective media. It was also likely that the mutated *ftsW* allele was the dominant allele. However, the ability to restore all phenotypes of Δ*sepIVA* to those of a WT strain solely by complementation with a recombinant copy of *sepIVA* suggest that the mutated *ftsW* allele had no role to play in the observed phenotypes, and was unlikely to have affected the ability to generate a viable *sepIVA* mutant. The complete open reading frame for *sepIVA* was not deleted in our Δ*sepIVA* mutant and it was possible that a putative 174 aa long peptide retaining 155 aa from the N-terminus of SepIVA was formed due to a readthrough into the *hyg-sacB* replacement cassette. As we do see a septation defect in our mutant Δ*sepIVA* strain, this shorter hypothetical peptide was unlikely to have retained functions directly related to septation. However, we cannot rule out a second role for the N-terminal of SepIVA in distinct interactions with the cell division apparatus, which dictate the essentiality of *sepIVA* in mycobacteria. Thus, after considering various possibilities, including the potential role of growth media, suppressor mutations and a potential truncated SepIVA produced in the Δ*sepIVA* strain, we were unable to conclusively identify a sole defining reason for our ability to obtain a viable null mutant of *sepIVA* in contrast to the two previous reports of essentiality. However, despite the differences in reports of essentiality, the rest of our studies report similar phenotypes to the conditional mutants characterized in these previous studies, particularly the elongated cells and altered septation patterns. Thus, this work, along with the previous findings of Wu *et al*. [14] and Jain *et al*. [15], does identify a key role for *sepIVA* in septation and warrants further studies to decipher its precise role.

## Methods

### Bacterial strains and culture conditions

*M. smegmatis* mc^2^155 was cultured in tryptic soy broth (TSB) supplemented with Tween-80 (0.05 %) to prevent clumping. *E. coli* strains (TOP10 and HB101) were cultured in Luria–Bertani broth. All bacterial strains were cultured at 37 °C with agitation. Where required, the following antibiotics were used for selection: hygromycin (150 µg ml^−1^ for *E. coli* and 100 µg ml^−1^ for *M. smegmatis *) and kanamycin (50 µg ml^−1^ for *E. coli* and 25 µg ml^−1^ for *M. smegmatis *). When examining pellicle formation of *M. smegmatis* strains, cultures were diluted in Sauton’s media to OD_600_ 0.05, and were cultured in 24-well plates. Cultures were incubated at 37 °C, 5 % CO_2_, with pellicles examined following 4 days of incubation. For testing survival on Middlebrook media, 10 ml of serial 10-fold dilutions of the *M.smegmatis* strains were plated on 7H9 broth+1.5 % agar, 7H10 or 7H11 plates (performed in triplicate).

### Construction of mutant strains

All of the plasmids and phages utilized in this work are outlined in Table 1. Approximately 1 kb of the left and right flanking regions of *MSMEG2416* were PCR-amplified from *M. smegmatis* mc^2^155 genomic DNA using the primer pairs MS2416_LL (5′-TTTTTTTTCCATAAATTGGCCTCGAAGAGGAACACAAG-3′) and MS2416_LR (5′-TTTTTTTTCCATTTCTTGGGATGTTGCCGTTCTCGATG-3′), and MS2416_RL (5′-TTTTTTTTCCATAGATTGGTTCGTGGCGAGTGCGACATC-3′) and MS2416_RR (5′-TTTTTTTTCCATCTTTTGGGATGGCGGTGGTACAGCTCTTC-3′). Van91I restriction sites were incorporated into the 5′ end of each primer. The resultant PCR products were digested with Van91I and cloned into Van91I-digested p0004s. Recombinant plasmids were verified by Van91I digestion and sequencing. The sequence confirmed plasmid was linearized with PacI and cloned into PacI-digested phAE159. The resultant phΔ*MSMEG2416* phasmid DNA was used to generate high-titre phage particles, and specialized transduction was performed as described previously [19]. *M. smegmatis* Δ*sepIVA* was confirmed by Southern blot analysis and whole-genome sequencing.

**Table 1.** List of bacterial strains, phages and plasmids used in this work

	Description	Source
**Bacterial strains**		
WT	* M. smegmatis* mc^2^ 155	[29]
Δ*sepIVA*	* M. smegmatis* mc^2^ 155 in which *sepIVA* (*MSMEG2416*) is replaced with *hyg*	This work
Δ*sepIVA*-C	Complemented strain of Δ*sepIVA*, containing pMV306-*MSMEG2416*	This work
Δ*sepIVA-*CRv	Complemented strain of Δ*sepIVA*, containing pMV306-*Rv2927c*	This work
**Plasmids**		
p0004s	Allelic exchange substrate vector containing *hyg*	[19]
pΔ*MSMEG_2416*	p0004s derivative constructed for the allelic exchange of *sepIVA* (*MSMEG_2416*)	This work
pMV306	Integrative *E. coli–Mycobacterium* shuttle vector; kan^r^	[27]
pMV306-*MSMEG2416*	pMV306 containing *MSMEG2416* with its native promoter	This work
pMV306-*Rv2927c*	pMV306 containing *Rv2927c* with its native promoter	This work
**Phages**		
phAE159	Temperature-sensitive derivative of mycobacteriophage TM4	[30]
phΔ*MSMEG2416*	phAE159 derivative designed for replacing *sepIVA* (*MSMEG2416*) with *hyg*.	This work

*MSMEG2416* and *Rv2927c*, and their native promoters, were PCR-amplified from *M. smegmatis* mc^2^155 and *M. tuberculosis* H37Rv genomic DNA, respectively, using the primer pairs MS2416_F (5′-GCGTCTAGAGATCGCGCCGACGCGTCCGC-3′) and MS2416_R (5′-GCGAAGCTTGCGGTGCTTCGCCGCGTTCG-3′), and Rv2927c_F (5′-GCGTCTAGACACGCTCGGGCAAGATCGCC-3′) and Rv2927c_R (5′-GCGAAGCTTGGCCATAAGAGAAATCCTAC-3′). Digested PCR products were cloned into the digested integrative vector, pMV306 [27]. The resultant plasmids were verified by digestion and sequencing (Table 1). Sequence-confirmed plasmids were electroporated into *M. smegmatis* mc^2^155 Δ*sepIVA*. Transformants were screened by selection on kanamycin and hygromycin plates, and confirmed as complemented strains by PCR. One such transformant complemented with *MSMEG_2416* (*sepIVA*) was termed Δ*sepIVA*-C and one with Rv2927c was termed Δ*sepIVA*-CRv. Similarly, to test the ‘poisoning’ effect of WT *ftsW* on the *sepIVA* mutant, *M. smegmatis ftsW* was PCR-amplified with its native promoter sequence and cloned into pMV306. The plasmid was transformed into both WT *M. smegmatis* mc^2^155 and Δ*sepIVA*.

### Microscopic examination of *M. smegmatis* strains

When measuring cell lengths, cells from mid-exponential phase cultures were visualized using a Nikon A1R inverted confocal microscope. A random sample of 250 individual cells were measured (visualized from randomly selected regions of the slides). Statistical analysis of cell lengths was performed with a two-way analysis of variance (ANOVA), using Bonferroni for multiple comparisons. *, *P*<0.05. For fluorescence microscopy, chromosomal material was visualized by staining with propidium iodide, and newly synthesized peptidoglycan in septa was stained using fluorescent vancomycin in the same way as described for *Streptomyces* [13], using mid-exponential phase cutlures. Staining of nascent peptidoglycan was performed by incubating growing cells using 2 µg ml^−1^ BODIPY FL vancomycin (Molecular Probes) and 2 µg ml^−1^ unlabelled vancomycin (Sigma), together with propidium iodide (Sigma, 10 µg ml^−1^) as described for *Streptomyces* [13]. Samples stained using fluorescent vancomycin were not fixed; and while propidium iodide is not expected to stain live, non-fixed samples, we found that 10 µg ml^−1^ propidium iodide routinely stained chromosomes of live *M. smegmatis* cells as well as cells of *Streptomyces *. Samples were viewed using a Zeiss Axioplan 2 microscope with an AxioCamMR camera and 100×1.4 NA Ph3 objective.

## Supplementary Data

Supplementary material 1
