# Supplementary material for: A mycobacterial DivIVA domain-containing protein involved in cell length and septation
Source: Microbiology (Reading). 2020 Jul 17;166(9):817–25. doi: 10.1099/mic.0.000952 (PMC7654743; doi:10.1099/mic.0.000952)
Supplement: Supplementary material 1 [file mic-166-817-s001.pdf]

Map of genomic region

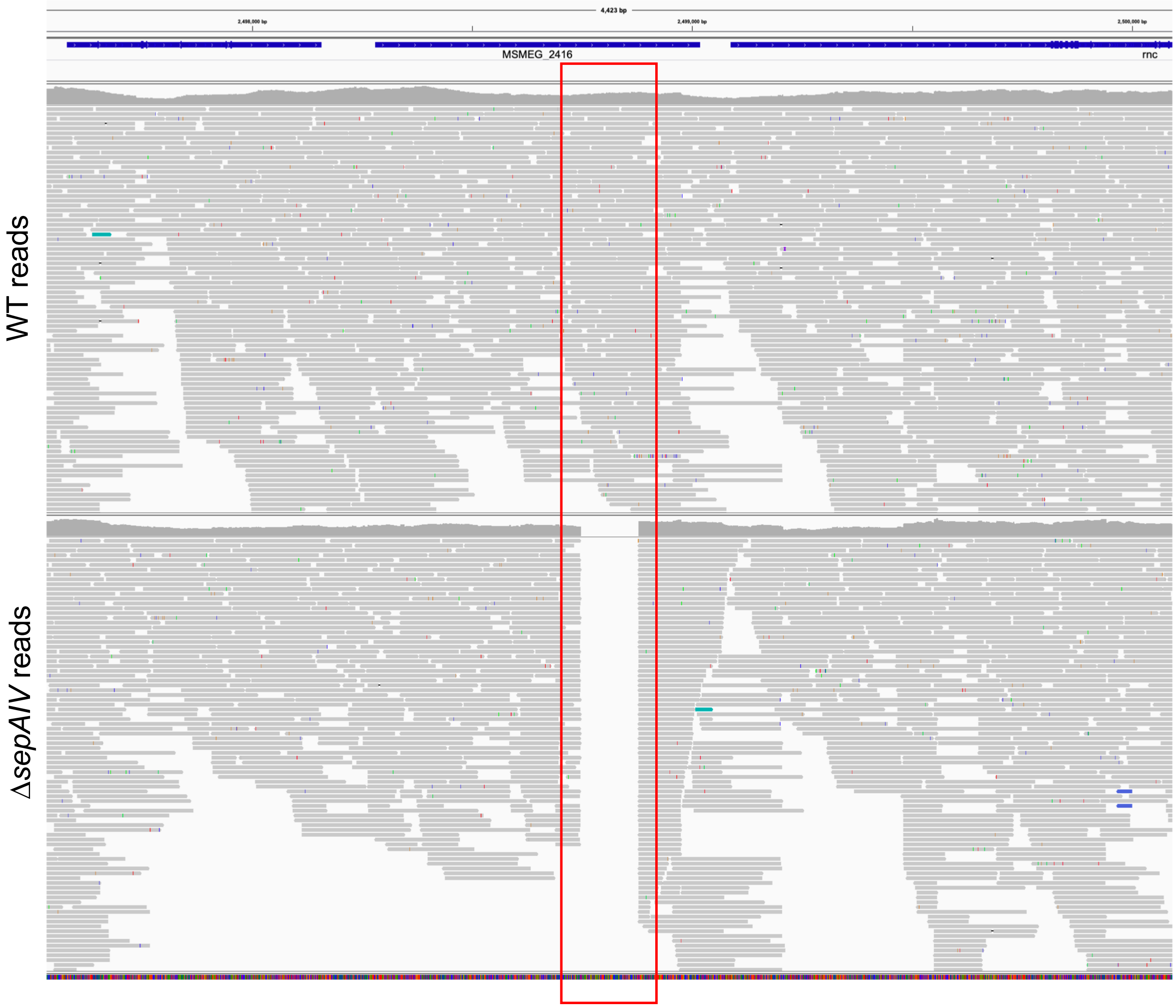

Supporting data Fig.1: Confirmation of generation of a *M. smegmatis* *sepIVA* null mutant by whole genome sequencing. Alignments of sequence reads of WT and Δ*sepIVA* mutant strains with *M. smegmatis* mc^{2}155 reference sequence. There is a clear lack of alignment of the reads from the Δ*sepIVA* mutant to a defined section of *sepIVA* (red box) that corresponds to the deleted portion of the gene following Specialized Transduction. A BLASTN alignment of the contig covering the *sepIV* region confirmed this result and also showed that the non-aligned sequences in the Δ*sepIVA* reads shown here matched with that of the *hyg-sacB* cassette (not shown).

(A)

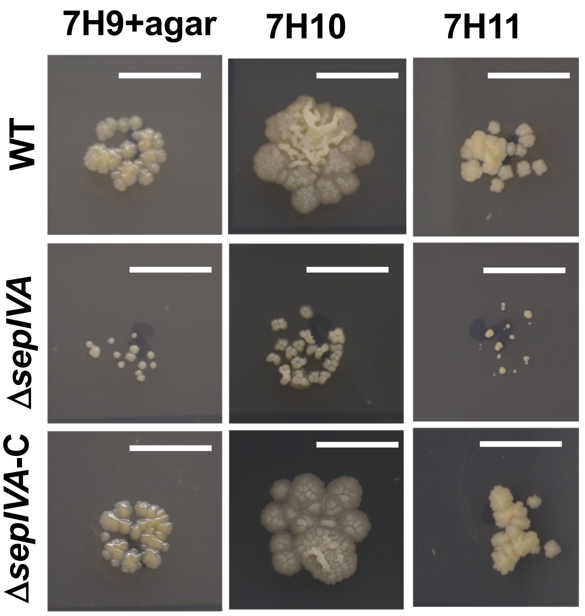

(B)

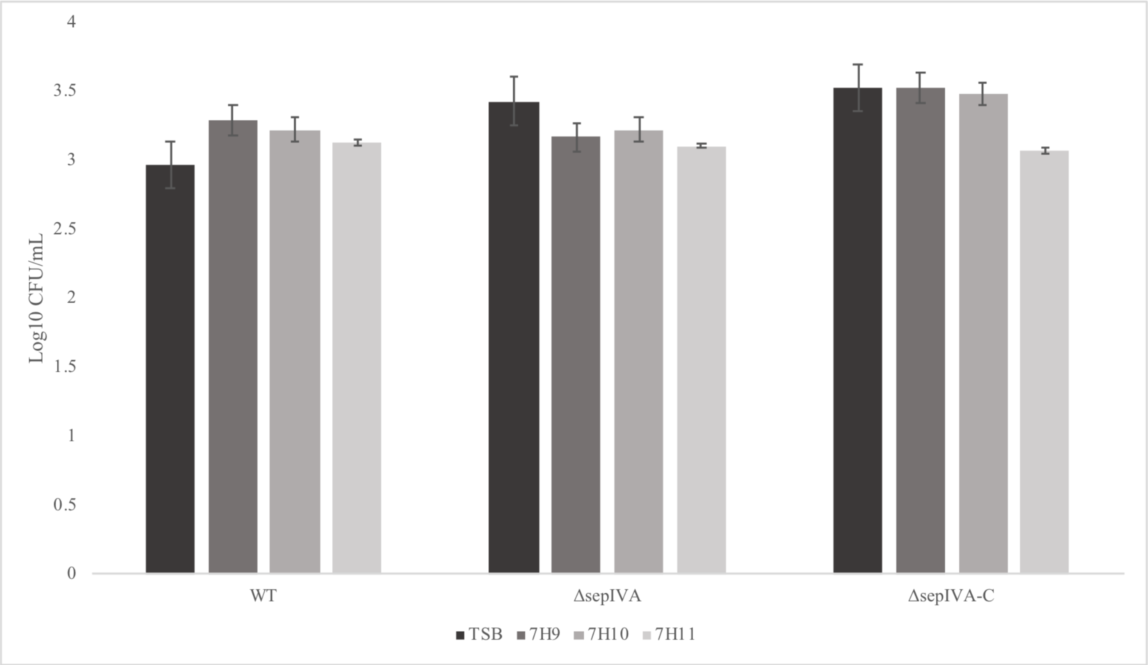

Supporting data Fig.2: Choice of media affected colony size, but not plating efficiency of the *M. smegmatis* ΔsepIVA. (A) Single colonies of WT and Δ*sepIVA* and complemented strains on Middlebrook media. Scale bar represents 1cm.(B) Average counts of the same diluted culture of each strain spotted on TSB agar and Middlebrook media plates after incubation at 37ºC for 5 days.

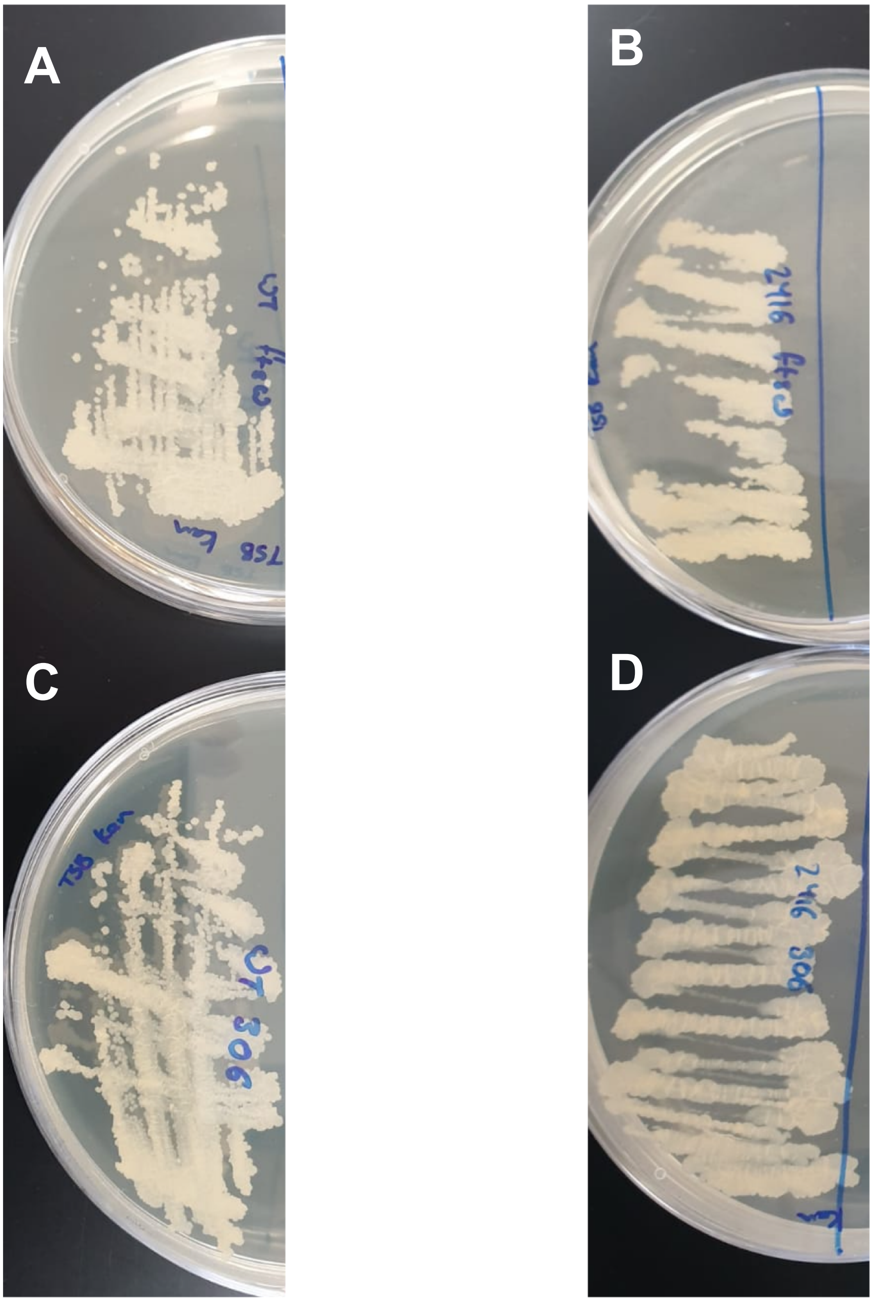

Supporting data Fig.3: *M. smegmatis* WT (A, C) or Δ*sepIVA* (B, D) strain transformed with wild type *ftsW* cloned in pMV306 *.* (A, B) or empty vector (C,D). Streaks obatined on kanamycin containing TSB agar plates after incubation at 37ºC for 5 days.
